# Nasal Myiasis in an Immunocompetent Child With Cerebral Palsy: A Case Report

**DOI:** 10.7759/cureus.90396

**Published:** 2025-08-18

**Authors:** Mohamed Bouallou, Mohammed Amine Rabhi, Achraf Sbai, Drissia Benfadil, Azzedine Lachkar, Fahd El Ayoubi

**Affiliations:** 1 Department of Otolaryngology, Head and Neck Surgery, Mohammed VI University Hospital Center, Oujda, MAR; 2 Department of Otolaryngology, Head and Neck Surgery, CHU Mohammed VI, Oujda, MAR; 3 Department of Otolaryngology, Head and Neck Surgery, Faculty of Medicine and Pharmacy, Mohammed First University, Oujda, MAR

**Keywords:** children, immunocompetent, lucilia, maggots, nasal myiasis

## Abstract

Myiasis is a parasitic infestation, either accidental or obligatory, that affects animals and humans and is caused by the larvae of flies, also known as maggots. Nasal myiasis is more commonly encountered in tropical and subtropical regions. We report the case of a five-year-old child with cerebral palsy residing in a rural area, with a history of contact with dogs and sheep, who was admitted to the otorhinolaryngology department after his parents noticed several larvae in his right nostril for the past two days. Clinical examination revealed maggots in the right nostril, while the nasopharynx and oral cavity were free of infestation. The maggots were manually removed using forceps under endoscopic visualization and were sent to the parasitology department for precise identification. Microscopic examination of the larvae confirmed the diagnosis of myiasis caused by *Lucilia*. The patient's nose was washed with a 2% solution of xylocaine, and 2 mg (0.2 mg/kg) of ivermectin tablets was given orally, and repeated after 24 hours. Subsequently, the patient's nasal symptoms resolved completely.

## Introduction

Myiasis, a term first introduced by William Hope in 1840, describes the infestation of tissues and body cavities of living vertebrate hosts by the eggs or larval stages of dipteran flies. The term “myiasis” comes from the Greek word “muia” or “mya” meaning fly and “iasis” meaning disease [1]. Various species of flies can induce myiasis in the head and neck region, exhibiting differing degrees of invasiveness and pathogenicity [2].

The most significant family implicated in myiasis is the Calliphoridae, which includes the genera *Lucilia*, *Calliphora*, *Chrysomyia*, and *Cochliomyia* [3]. The first report of myiasis caused by *Lucilia* *sericata* dates back to 1826, when Magen extracted maggots from the eyes, mouth, and paranasal sinuses of a hospitalized patient [4]. Nasal myiasis due to *Lucilia sericata* is infrequently reported in the literature. In Morocco, the fly family most commonly implicated in cases of myiasis is the Oestridae [5].

The disease affects both genders equally and predominantly manifests in individuals aged over 50 years. Nasal myiasis commonly presents with symptoms such as epistaxis, nasal obstruction, fetid rhinorrhea, facial pain, and headache.

The diagnosis of nasal myiasis is primarily based on thorough history-taking and physical examination. Nasal myiasis is an uncommon condition, with only a limited number of reported cases and no established consensus on treatment [6].

We present the case of a five-year-old immunocompetent child with cerebral palsy, residing in a rural area and exhibiting poor overall hygiene, who was admitted for management of nasal myiasis.

## Case presentation

**Figure 1:**
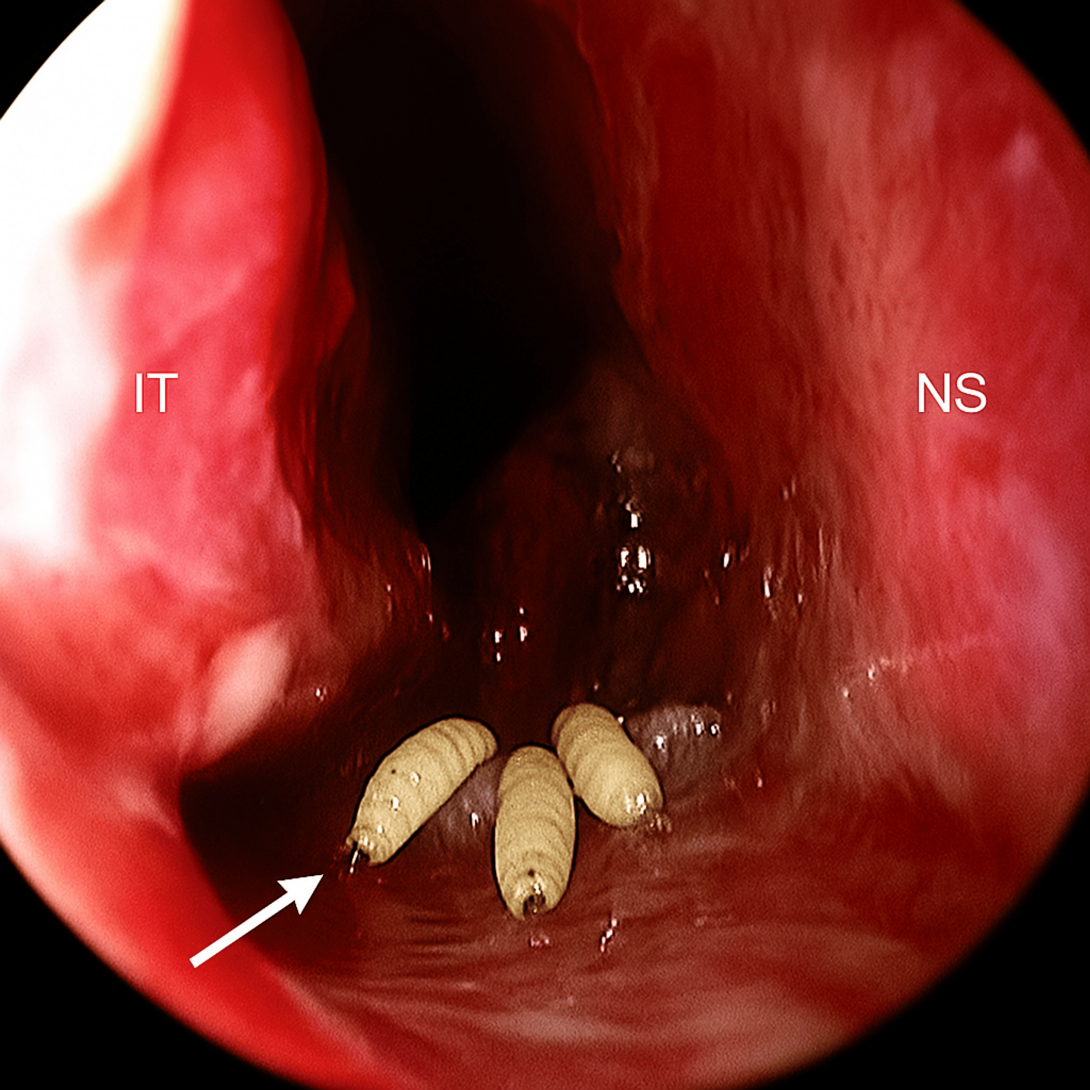
Rhinoscopy revealed multiple larvae in the right nostril (with arrow) IT: inferior turbinate, NS: nasal septum

**Figure 2:**
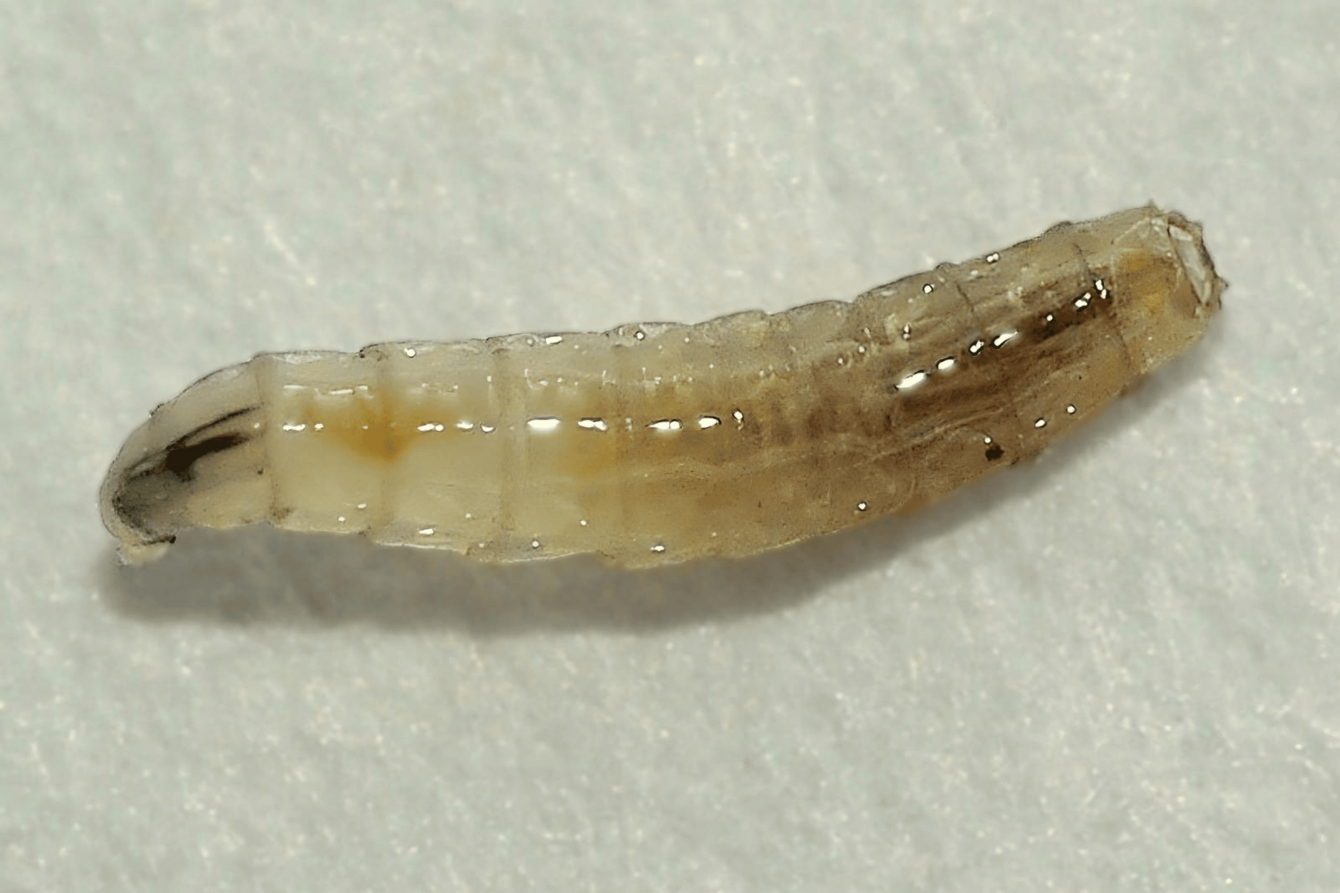
Maggots after extraction with forceps

A five-year-old male patient with cerebral palsy residing in a rural area, with a history of contact with dogs and sheep, was admitted to the otorhinolaryngology department after his parents noticed several larvae in his right nose for the past two days. Clinical examination revealed a conscious child (Glasgow Coma Scale (GCS) 15/15), stable from a respiratory standpoint (oxygen saturation: 99%), and hemodynamically stable (blood pressure: 120/75 mmHg), afebrile (temperature: 37.1°C). Nasofibroscopy of the patient’s nasal cavity revealed erythematous but non-inflamed mucosa, absence of turbinate hypertrophy, patent nasal meatuses, no crusting, and no septal deviation. Maggots were observed in the right nostril, while the contralateral side appeared normal (Figure 1). The examination revealed no maggots in the nasopharynx and oral cavity. The otoscopic examination was normal. Palpation of the neck did not reveal any cervical lymphadenopathies. The abdominal examination revealed no abnormalities. The remainder of the clinical examination was unremarkable. There was evidence of poor general hygiene. The maggots were manually removed using forceps under endoscopic visualization, followed by regular nasal irrigation, and were sent to the parasitology department for precise identification. Microscopic examination of the larvae confirmed the diagnosis of myiasis caused by *Lucilia*. The specimen measured 13 mm in length (Figure 2). The patient's nose was washed with a 2% solution of xylocaine, and 2 mg (0.2 mg/kg) of ivermectin tablets was given orally, and repeated after 24 hours.

Subsequently, the patient's nasal symptoms resolved completely. The patient was discharged after five days and followed up six weeks later. No more larvae were observed in the nasal cavity, and no recurrence of myiasis was noted on follow-up.

## Discussion

Nasal myiasis is a common condition in tropical, subtropical, and developing countries [7,8], where warm weather and humidity provide an excellent environment for this infestation. The incidence of myiasis tends to increase between March and June [9]. Nasal myiasis is often considered an accidental nasal infestation. Myiasis commonly affects mammals; however, in humans, it is more frequently observed in rural areas where people often have direct contact with animals [10], as was the case with our patient. It is usually more prevalent among individuals from lower socioeconomic groups.

Different types of myiasis, including nasal myiasis, have been reported in the literature. However, cases of nasal myiasis are rarely reported in the country. Most myiases are generally caused by species of the Oestridae family, particularly *Oestrus ovis* [11]. Most cavitary myiases are caused by *Lucilia* [5]. In Morocco, to date, the sole documented case of nasal myiasis attributed to *Lucilia* was reported in 2022, involving a 72-year-old patient who had been admitted to the intensive care unit for the management of septic shock secondary to complicated peritonitis [5].

Human myiasis is caused by flying dipteran insects that lay their eggs on healthy or necrotic tissues, infected and foul-smelling wounds, or occasionally within the nasal, oral, or external auditory canal [12]. The development of larvae from deposited eggs largely depends on environmental factors such as humidity and temperature [13]. These flying dipterans are attracted to animals by the scent of urine and feces. After deposition, the larvae penetrate the tissues using their cephalopharyngeal skeleton apparatus and secretion of proteolytic enzymes [3].

Nasal myiasis is primarily favored by open wounds, patient immobilization, infections, underlying systemic diseases, and any conditions leading to immunosuppression, as well as poor hygienic conditions often seen in patients with mental disability [14]. In our case, the presence of cerebral palsy associated with poor hygienic conditions served as the principal predisposing factor, as the child’s disability limited his ability to repel flying insects.

The diagnosis of nasal myiasis is primarily clinical, relying on characteristic symptoms and the direct identification of larvae within the nasal cavity. Generally, nasal myiasis is a benign condition that most commonly presents with epistaxis, foul odor, pain, and a sensation of foreign bodies within the nasal cavity [7]. However, our case was asymptomatic; the condition was incidentally discovered when a maggot was expelled during a sneezing episode, which prompted the parents to seek medical consultation.

The conventional treatment of myiasis involves the manual removal of all larvae, performed either directly or under endoscopic guidance. However, no standardized treatment protocol exists for nasal myiasis [15]. Several substances have been employed to facilitate larval removal, including instillation of a mixture of chloroform and turpentine oil, ethylene chloride, irrigation with naphtha, ether, cocaine, ivermectin 1%, and lidocaine [16]. In our case, we used lidocaine and oral ivermectin. Furthermore, oral ivermectin has been reported in the literature for the treatment of orbital and oral myiasis. A single dose of 0.2 mg/kg was administered in a case of orbital myiasis as described [17]. Ivermectin acts by enhancing the release and receptor affinity of gamma-aminobutyric acid (GABA) at the synapses of endoparasitic nematodes, which activates GABA-gated chloride channels. This results in increased intracellular chloride levels, causing hyperpolarization, paralysis, and ultimately death of the parasites [16]. Endoscopic examination performed three days later revealed no remaining larvae.

## Conclusions

Nasal myiasis has become an infrequent occurrence in humans due to improved living conditions; nevertheless, it continues to be reported in developing and underdeveloped countries. In our case, poor hygiene combined with cerebral palsy constituted significant predisposing factors facilitating the development of maggot. Indeed, the endoscopically guided extraction of maggots, combined with thorough nasal irrigation, topical administration of lidocaine, and systemic treatment with ivermectin, yielded satisfactory and prompt clinical improvement.

Our report highlights several important points, emphasizing the critical role of sanitation and hygiene in rural areas in preventing myiasis. The installation of fine mesh screens on windows to exclude insects can serve as an effective and efficient preventive measure against myiasis.

## References

[1] Rana AK, Sharma R, Sharma VK, Mehrotra A, Singh R (2020). Otorhinolaryngological myiasis: the problem and its presentations in the weak and forgotten. Ghana Med J.

[2] Duque C, Marrugo G, Valderrama R (1990). Otolaryngic manifestations of myiasis. Ear Nose Throat J.

[3] SA S, RA M, MA S, MA F (2018). Nasal myiasis: a case report. Iran J Public Health.

[4] Daniel M, Šrámová H, Zalabska E (1994). Lucilia sericata (Diptera: Calliphoridae) causing hospital-acquired myiasis of a traumatic wound. J Hosp Infect.

[5] Bouikhif M, El Kettani Y, Lyagoubi M, Aoufi S (2022). Nasal myiasis due to lucilia sp. in intubated patient: about one case in Morocco. Med Trop Sante Int.

[6] White ZL, Chu MW, Hood RJ (2015). Nasal myiasis: a case report. Ear Nose Throat J.

[7] Kim JS, Seo PW, Kim JW, Go JH, Jang SC, Lee HJ, Seo M (2009). A nasal myiasis in a 76-year-old female in Korea. Korean J Parasitol.

[8] Basmaciyan L, Gabrielle PH, Valot S, Sautour M, Buisson JC, Creuzot-Garcher C, Dalle F (2018). Oestrus ovis external ophtalmomyiasis: a case report in Burgundy France. BMC Ophthalmol.

[9] Swain SK, Sahu MC, Baisakh MR (2018). Nasal myiasis in clinical practice. Apollo Med.

[10] Ahmad AK, Abdel-Hafeez EH, Makhloof M, Abdel-Raheem EM (2011). Gastrointestinal myiasis by larvae of Sarcophaga sp. and Oestrus sp. in Egypt: report of cases, and endoscopical and morphological studies. Korean J Parasitol.

[11] Tligui H, Oudaina W, Khairane I (2011). Rhino-myiase humaine à Oestrus ovis: une observation au Maroc. Med Trop.

[12] Mowlavi G, Nateghpour M, Teimoori S, Amin A, Noohi F, Kargar F (2011). Fatal nosocomial myiasis caused by Lucilia sericata. J Hosp Infect.

[13] Olatoke F, Afolabi OA, Lasisi OA, Alabi BS, Aluko AA (2011). Aural myiasis: case report from Nigeria. Int J Pediatr Otorhinolaryngol Extra.

[14] Kumar P, Singh V (2014). Oral myiasis: case report and review of literature. Oral Maxillofac Surg.

[15] Surayya R, Parwati DR (2021). Management of nasal myiasis and type 2 diabetes mellitus: a rare case and review article. Int J Surg Case Rep.

[16] Sayeed A, Ahmed A, Sharma SC, Hasan SA (2019). Ivermectin: a novel method of treatment of nasal and nasopharyngeal myiasis. Indian J Otolaryngol Head Neck Surg.

[17] De Tarso P, Pierre-Filho P, Minguini N, Pierre LM, Pierre AM (2004). Use of ivermectin in the treatment of orbital myiasis caused by Cochliomyia hominivorax. Scand J Infect Dis.

